# Linkages between Chiropteran diversity and ecosystem services for sustainable fragmented forest conservation

**DOI:** 10.1016/j.dib.2018.11.058

**Published:** 2018-11-14

**Authors:** Siti Nurfatiha Najihah Fakhrul-Hatta, Bryan Raveen Nelson, Nur Juliani Shafie, Muhamad Aidil Zahidin, Mohd. Tajuddin Abdullah

**Affiliations:** a School of Marine and Environmental Sciences, Universiti Malaysia Terengganu, 21030 Kuala Nerus, Terengganu, Malaysia; b Institute of Tropical Biodiversity and Sustainable Development, Universiti Malaysia Terengganu, 21030 Kuala Nerus, Terengganu, Malaysia

## Abstract

This data article informs about Chiropteran diversity, new records, ecosystem services and possible pathogen carriers in fragmented forests (sub-divided by utility corridors, man-made structures, untouched and secondary plantations) within districts Setiu (Setiu Research Station), Hulu Terengganu (Saok and Lasir waterfalls) and Besut (Gunung Tebu Forest Reserve) of state Terengganu, Peninsular Malaysia. These bats were captured using harp traps and mist nets that were set 10 m apart across flyways, streams and less cluttered trees in the 50 m × 50 m transect zones (identified at each site). All animals were distinguished by morphology and gender before their release at the site of capture. The data comprise of five bat family groups Hipposideridae, Megadermatidae, Pteropodidae, Rhinolophidae and Vespertilionidae. It is interesting to note that untouched Saok Waterfalls is home to wide variety of bats listed (68.8%), followed by secondary forests of Gunung Tebu Forest Reserve (24.8%), untouched Lasir Waterfalls (4.8%) and lastly, Setiu Research Station as least favored (1.6%). Chiroptera like *Cynopterus brachyotis* (*n* = 23, 37.7%), *Hipposideros bicolor* (*n* = 6, 9.8%) and *Scotophilus kuhli* (*n* = 6, 9.8%) were most dominant in the checklist whereas *Hipposideros armiger*, *Murina suilla* and *Scotophilus kuhlii* are new data records in the fragmented forests of Terengganu. The data were interpret into Shannon, Simpson, Margalef, Menhinik and Evenness indices to individually or collectively distinguish chiropteran variety in Terengganu State whereas weight-forearm length (W/FA) informs about chiropteran Body Condition Index (-0.25 to 0.25). The function of bats were also identified to distinguish service providers (pollination and forests regeneration) and zoonotic pathogen carriers (in particular to Leptospira bacteria, Nipah virus and Sindbis virus).

**Specifications table**

**Table:** 

Subject area	*Biology*
More specific subject area	*Bioscience and Biodiversity*
Type of data	*Tables*
How data was acquired	*Four bank harp trap (4.2 m*^*2*^*) and mist nets (Height = 2.6 m and Width = 10 m), Vernier caliper (sensitivity 0.1 cm), measuring tape, analytical balance (sensitivity 0.1 kg) and Paleontological Statistics Software Package (PAST) v.3*
Data format	*Raw and Analyzed*
Experimental factors	*Harp trap and net placement at flyways, streams and less cluttered trees and, the 10 m spacing between the traps and nets.*
Experimental features	*Descriptive abundance by pool, ecosystem services, pathogen-carrying species, guild types, roost, climb limit and range. Interpretations include body condition index and diversity estimations using Shannon, Simpson, Evenness, Margalef and Menhinick Indices.*
Data source location	*1. Setiu District, Terengganu, East Peninsular Malaysia*
	*Setiu Wetland Research Centre: N 5.6771°; E 102.7102°*
	*2. Hulu Terengganu District (Terengganu Nature Park), Terengganu, East Peninsular Malaysia*
	*Lasir Waterfalls: N 4.9655°; E 102.8396°*
	*Saok Waterfalls: N 5.0832°; E 102.7784°*
	*3. Besut District, Terengganu, Peninsular Malaysia*
	*Gunung Tebu Forest Reserve: N 5.5915°; E 102.6164°*
Data accessibility	*All raw data are available within this article*
Related research article	*Unpublished data*

**Value of the data**

• This data is constructed from chiropteran diversity and abundance in fragmented forests of Terengganu with some new records that offer research opportunities and perhaps collaboration to address the subject matter.
• The chiroptera discovered in the present study are ecological service providers that maintain tropical rainforest splendor, influence the genetic vigor of fruits, flora and timber, become pest controllers, function as pathogen reservoirs and indirectly assist agriculture industry through crop pollination, seed dispersal and provide nutrient-rich feces as manure. These form of valuable information provides insights into forest and wildlife management, serve as benchmark for fragmented forests and as indicator for climate change, assist with food web plotting in different environments (caves, trees, artificial structures and fragmented forests) and also assist with bio-resource studies (like biotechnology and phenology).
• Chiropteran morphometric data translated into body condition index changes the research perspective because wildlife stress, forest carrying capacity and forest regeneration are measurable. These measurements can be incorporated into prediction models used for sustainability and conservation studies.

## 1 Data

This data article is constructed using survey results that indicate bat diversity and their abundance in fragmented forests within Terengganu (Table 1). From these, additional associations such as new records, ecosystem services, possible pathogen carriers and their lifestyle were added from available literature for forest regeneration impacts, influence towards agriculture activities, trophic level and food web constructs, natural pest control, precursor for diseases, conservation assert (if needed) as well as their geographical tolerance and adaptations (Table 2). Chiropteran morphometric measurements are translated into weight-length data to scale their health in the wild (Table 3). These indicate food resources, its availability and its adaptation to forest fragmentations. The data is also interpret using Mathematical equations (Shannon, Simpson, Eveness, Margalef and Menhinick) to derive diversity and species richness values which are useful for chiropteran interpolations and assessments within a geographical boundary (Table 4).

**Table 1:** Chiropteran abundance from sites assessed in the districts Setiu, Besut and Hulu Terengganu, Peninsular Malaysia.

Family	Species	Common name	Abundance (No.)			
			SW	LW	GTFR	SRS
Hipposideridae	^*N*^*Hipposideros armiger*	Great Leaf-nosed Bat	0	0	2	0
	*Hipposideros bicolor*	Bicoloured Leaf-nosed Bat	5	0	1	0
	*Hipposideros cervinus*	Fawn Leaf-nosed Bat	2	1	0	0
	*Hipposideros diadema*	Diadem Leaf-nosed Bat	0	0	1	0
Megadermatidae	*Megaderma lyra*	Greater False Vampire Bat	0	0	1	0
Pteropodidae	*Balionycteris maculata*	Spotted-winged Fruit Bat	0	0	1	0
	*Cynopterus brachyotis*	Lesser Dog-faced Fruit Bat	21	1	0	1
	*Cynopterus horsefieldii	Horsefield’s Fruit Bat	3	0	0	0
	*Eonycteris spelaea*	Cave Nectar Bat	1	0	0	0
	*Macroglossus sobrinus*	Long-tongued Fruit Bat	3	0	0	0
	*Megaerops ecaudatus*	Tailless Fruit Bat	1	0	0	0
	*Penthetor lucasi*	Dusky Fruit Bat	2	1	0	0
Rhinolophidae	*Rhinolophus affinis*	Intermediate Horseshoe Bat	1	0	3	0
	*Rhinolophus lepidus*	Blyth’s Horseshoe Bat	2	0	0	0
Vespertilionidae	^*N*^*Murina suilla*	Brown Tube-nosed Bat	1	0	0	0
	^*N*^*Scotophilus kuhlii*	Lesser Asiatic Yellow House Bat	0	0	6	0
Total			42	3	15	1

Note: The status of all bats are ‘Least Concern’ unless marked with * to indicate it as ‘Not Evaluated’ in the IUCN Red List. New records are shown with ‘N’ for their absence even in the most recent study by Pounsin et al. [1]. Sites for field visits are abbreviated as SW = Saok Waterfalls, LW = Lasir Waterfalls, GTFR = Gunung Tebu Forest Reserve and SRS = Setiu Research Station. Abundance of bats are denote with ‘no.’ to represent number of individuals.

**Table 2:** Mean forearm length and weight of voucher Chiropteran used to construct the weight to length ratio and the comparisons used to construct the mass index.

Species	Forearm length (mm)		Weight (g)		W/FA (%)		Repository	BC Index	
	Male	Female	Male	Female	Male	Female	W^w^/FA^w^	Male	Female
*Hipposideros armiger*	91.2 ± 0.0	89.6 ± 0.0	68.0 ± 0.0	49.1 ± 0.0	74.5	54.8	48.0	0.6^E^	0.1 ^H^
*Hipposideros bicolor*	43.4 ± 1.3	46.0 ± 0.0	8.1 ± 0.9	5.8 ± 0.9	18.6	12.5	20.8	−0.1 ^H^	−0.4^U^
*Hipposideros cervinus*	47.0 ± 0.0	50.1 ± 0.0	9.4 ± 0.0	10.9 ± 0.0	20.0	21.7	20.0	0.0 ^H^	0.1 ^H^
*Hipposideros diadema*	83.6 ± 0.0	–	25.7 ± 0.0	–	30.7	–	54.7	−0.4^U^	–
*Megaderma lyra*	–	79.1 ± 0.0	–	52.4 ± 0.0	–	66.2	56.8	–	0.2 ^H^
*Balionycteris maculata*	–	44.0 ± 0.0	–	14.8 ± 0.0	–	33.7	34.1	–	0.0 ^H^
*Cynopterus brachyotis*	61.2 ± 4.4	61.8 ± 5.4	29.1 ± 9.4	32.0 ± 10.3	47.6	51.8	40.8	0.2 ^H^	0.3^E^
*Cynopterus horsefieldii*	75.9 ± 2.9	74.0 ± 0.0	49.8 ± 14.5	57.9 ± 0.0	94.8	78.2	92.1	0.0 ^H^	−0.2 ^H^
*Eonycteris spelaea*	–	68.0 ± 0.0	–	44.2 ± 0.0	–	65.0	79.3	–	−0.2 ^H^
*Macroglossus sobrinus*	45.3 ± 0.6	–	16.2 ± 4.6	–	35.7	–	44.3	−0.2 ^H^	–
*Megaerops ecaudatus*	54.0 ± 0.0	–	24.1 ± 0.0	–	44.6	–	44.3	0.0 ^H^	–
*Penthetor lucasi*	54.0 ± 0.0	56.1 ± 0.0	26.7 ± 0.0	27.9 ± 0.0	49.4	49.7	71.0	−0.3^U^	−0.3^U^
*Rhinolophus affinis*	51.0 ± 3.6	52.2 ± 0.0	13.5 ± 1.8	14.1 ± 0.0	26.5	27.0	27.7	0.0 ^H^	0.0 ^H^
*Rhinolophus lepidus*	42.5 ± 0.0	–	15.9 ± 0.0	–	37.4	–	13.4	1.8^B^	–
*Murina suilla*	–	31.5 ± 0.0	–	4.0 ± 0.0	–	12.7	14.0	–	−0.1 ^H^
*Scotophilus kuhlii*	50.8 ± 0.0	51.0 ± 1.4	19.6 ± 0.0	20.3 ± 2.7	38.6	39.9	42.3	−0.1 ^H^	−0.1 ^H^

Note: Weight to forearm length (W/FA) measurements were recorded from voucher chiropteran and compared with known measurements (W^w^/FA^w^) to construct the Body Condition Index (BCI). Values in this index were scaled by 0.5 unit differences into U = Underweight (−0.75 to −0.25), H = Ideal (-0.25 to 0.25), E = Overweight (0.25 to 0.75) and B = Obese (>0.75). Symbol – indicates absence of samples.

**Table 3:** Chiropteran ecological services, possible pathogen associations and limitations in the fragmented forests of Terengganu.

Species	Service	Location			Possible pathogen associations									Guild	Roost	Climb Limit (× 10^3^ m)	Range
	P	Pr	R	S	CI	IK	J	JE	L	N	PP	Sb	Sc				
*Hipposideros armiger*				/				/				/		AI	Cv	2.0	N
*Hipposideros bicolor*			/									/		AI	Cv	0.6	E^2^
*Hipposideros cervinus*		/	/						/			/		AI	Cv, T	1.4	N
*Hipposideros diadema*		/	/									/		AI	Cv, T	1.2	N
*Megaderma lyra*				/										C, I	B, Cv	1.0	E^1^
*Balionycteris maculata*	/		/						/					F, N	Cv	1.5	E^3^
*Cynopterus brachyotis*	/		/		/	/	/		/	/	/			F, N	Cv, T	1.6	N
*Cynopterus horsefieldii*	/			/										F, N	Cv, T	>1.0	E^2^
*Eonycteris spelaea*	/			/		/				/	/			N	Cv	1.0	N
*Macroglossus sobrinus*	/			/										N	T	2.0	N
*Megaerops ecaudatus*	/		/											F, N	Fs	3.0	E^2^
*Penthetor lucasi*	/		/						/					F, N	Cv	0.6	E^2^
*Rhinolophus affinis*		/	/									/	/	AI	Cv	2.0	N
*Rhinolophus lepidus*		/	/			/						/		AI	Cv	2.3	E^1^
*Murina suilla*		/	/											AI	Fs, P	1.5	N
*Scotophilus kuhlii*				/		/				/				AI	B	1.1	E^1^

Note: Chiropteran service as pollinator is indicated with (P). The forest types are divided into Primary Forests (Pr), Regenerated Forests (R) and Secondary forests (S) (personal observations). Pathogens that associate with Chiropterans include Carey Island virus (CI), Jugra virus (J), Japanese Encephalitis virus (JE), Nipah virus (N), Phnom Penh Bat virus (PP), Sindbis virus (Sb), Issyk-Kul (Keterah Virus), Leptospira bacteria and SARS coronavirus (Sc). Chiroptera guilds are denote with AI = Aerial Insectivore, C = Carnivore, F = Frugivore, I = Insectivore and N = Nectarivore whereas their roost are denote with B = buildings, Cv = Cave, Fs = Forests, P = Plantations and T = Trees. Chiropteran climb limits were recorded as maximum climb height. The geographical range of bats are classified into E^1^ = Endemic to Indomalayan Realm; E^2^ = Endemic to Indonesia & Malaysia; E^3^ = Endemic to Malaysia and N = Native.

**Table 4:** Site-wise pooling of diversity indices to describe Chiroptera in Setiu, Hulu Terengganu and Besut districts.

Criteria		Locations			
		SW	LW	GTFR	SRS
Diversity Index	Shannon	1.77	1.01	1.68	–
	Simpson	0.72	0.67	0.76	–
	Evenness	0.53	–	0.77	–
Richness Index	Menhinick	1.70	1.73	1.80	–
	Margalef	2.68	1.82	2.22	–

The sites for voucher sample collection are indicated by SW = Saok waterfalls and LW = Lasir Waterfalls in Hulu Terengganu, GTFR = Gunung Tebu Forest Reserve in Besut and, SRS = Setiu Research Station in Setiu. Symbol – indicates absence of statistics subject to insufficient samples.

## 2 Experimental design, materials, and methods

Field visits to districts Hulu Terengganu (Saok and Lasir Waterfalls), Setiu (Setiu Research Station) and Besut (Gunung Tebu Forest Reserve) were carried out in June 2017 and spanned six days at each site. While four-bank harp and mist nets were used to trap the bats (chiropterans), these devices were constructed in open spaces between trees after adapting with procedures of Jayaraj et al. [2]. In consideration, the trees should have ten meter distances, a vital criteria to construct the 50 × 50 (2500 m^2^) transect. Entanglement and capture of bats were carried out hourly every day after sunset and before sunrise (between 1830 and 0630). All bats were measured for size and weight using Vernier caliper and portable analytical balance (sensitivity ± 0.01 kg). Specific measurements of bats such as ear, head-body, tail, forearm, tarsus and hind foot were used to distinguish the bat identity. Descriptive measurements of bats such as forearm length and weight were used to construct the Body Condition Index adopted from Suba et al. [3]. In the presence of negative values, the body mass scale was adapted with the values and separated by 0.5 differential margins that give rise to underweight, ideal, overweight and obese. Bat identity were associated with services, dwelling locations, possible pathogen associations, guilds and roosts, climb limits and range [4], [5]. All chiropteran were released at the site of capture after data acquisition. Recapture was prevented by excluding bats with same gender and size that have hair shavings on their hind foot. The data was interpret into diversity values of Shannon, Simpson, Evenness, Menhinick and Margalef using Paleontological Statistics Software Package (PAST) v.3 because these constructs accurately profile bats by abundance, diversity and richness at each site.
