# Supplementary material for: Linkages between Chiropteran diversity and ecosystem services for sustainable fragmented forest conservation
Source: Data Brief. 2018 Nov 14;21:2089–94. doi: 10.1016/j.dib.2018.11.058 (PMC6262155; doi:10.1016/j.dib.2018.11.058)
Supplement: Supplementary file 1 — Transparency document [file mmc1.doc]

Conflict of Interest and Authorship Conformation Form

Please check the following as appropriate:

( / ) All authors have participated in (a) conception and design, or analysis and interpretation of the data; (b) drafting the article or revising it critically for important intellectual content; and (c) approval of the final version.

( / ) This manuscript has not been submitted to, nor is under review at, another journal or other publishing venue.

( / ) The authors have no affiliation with any organization with a direct or indirect financial interest in the subject matter discussed in the manuscript

Author’s name Affiliation

1) Siti Nurfatiha Najihah Fakhrul Hatta School of Marine and Environment Sciences Universiti Malaysia Terengganu

2) Nur Juliani Shafie School of Marine and Environment Sciences Universiti Malaysia Terengganu

3) Muhamad Aidil Zahidin School of Marine and Environment Sciences Universiti Malaysia Terengganu

Institute of Tropical Biodiversity and Sustainable Development, Universiti Malaysia Terengganu

4) Bryan Raveen Nelson Institute of Tropical Biodiversity and Sustainable Development, Universiti Malaysia Terengganu

5) Mohd Tajuddin Abdullah School of Marine and Environment Sciences Universiti Malaysia Terengganu

Institute of Tropical Biodiversity and Sustainable Development, Universiti Malaysia Terengganu
